# Poly[[aqua-μ_6_-benzene-1,2,3-tricarboxyl­ato-μ_3_-hydroxido-dizinc] hemihydrate]

**DOI:** 10.1107/S1600536811055358

**Published:** 2012-01-07

**Authors:** Chao-Hong Ma, Qiang Liu, Xiu-Yan Wang, Xian-Wu Dong, Yu-Jie Li

**Affiliations:** a Jilin Agricultural Science and Technology College, Jilin 132101, People’s Republic of China

## Abstract

In the title compound, {[Zn_2_(C_9_H_3_O_6_)(OH)(H_2_O)]·0.5H_2_O}_*n*_, there are three independent Zn^II^ atoms present; two are located on special positions, *viz* a twofold rotation axis and an inversion centre, and the third is located in a general position. The Zn^II^ atom on the inversion centre is six-coordinated by four O atoms from four different benzene-1,2,3-tricarboxyl­ate anions and two OH^−^ anions. The Zn^II^ atom located on a twofold axis is four coordinated by two O atoms from two different benzene-1,2,3-tricarboxyl­ate anions and two OH^−^ anions. The third Zn^II^ atom, located in a general position, is five coordinated by three O atoms from three different benzene-1,2,3-tricarboxyl­ate anions, one OH^−^ anion and one water mol­ecule. Each benzene-1,2,3-tricarboxyl­ate anion bridges six Zn^II^ atoms, and the OH^−^ anion bridges three Zn^II^ atoms, resulting in the formation of a three-dimensional framework. A series of O—H⋯O hydrogen bonds involving the benzene-1,2,3-tricarboxyl­ate anions, the OH^−^ anion and the coordinating and the two water solvent mol­ecules further stablize the crystal structure. The two solvent water molecules show occupancies of 0.5 and 0.25.

## Related literature

For complexes of benzene tricarb­oxy­lic acids, see: Chui *et al.* (1999 ▶); Majumder *et al.* (2005 ▶). For related structures, see: Wu *et al.* (2009 ▶).
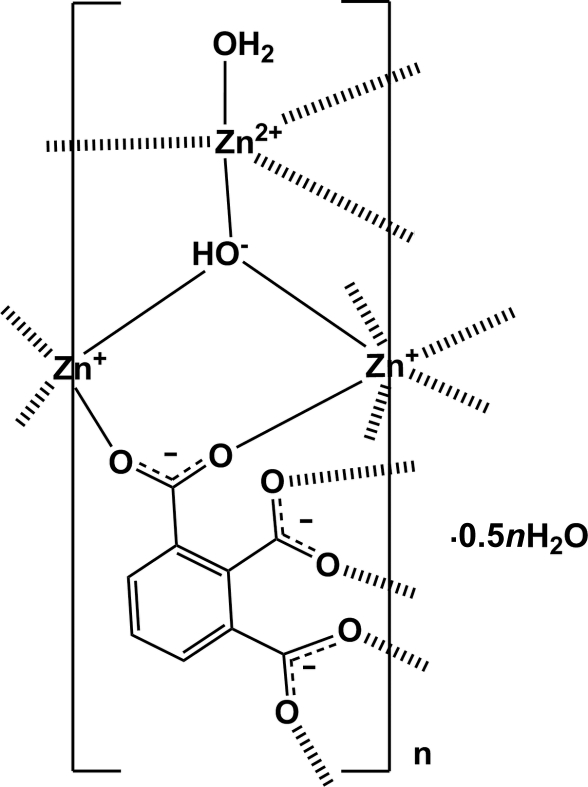


## Experimental

### 

#### Crystal data

[Zn_2_(C_9_H_3_O_6_)(OH)(H_2_O)]·0.5H_2_O
*M* *_r_* = 381.89
Tetragonal, 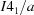

*a* = 12.8412 (2) Å
*c* = 27.2647 (7) Å
*V* = 4495.85 (15) Å^3^
*Z* = 16
Mo *K*α radiation
μ = 4.31 mm^−1^
*T* = 293 K
0.28 × 0.23 × 0.21 mm

#### Data collection

Oxford Diffraction Gemini R Ultra diffractometer
Absorption correction: multi-scan (*CrysAlis RED*; Oxford Diffraction, 2007 ▶) *T*_min_ = 0.312, *T*_max_ = 0.399
9288 measured reflections
2048 independent reflections
1402 reflections with *I* > 2σ(*I*)
*R*_int_ = 0.051

#### Refinement

*R*[*F* ^2^ > 2σ(*F* ^2^)] = 0.035
*wR*(*F* ^2^) = 0.090
*S* = 0.94
2048 reflections
198 parameters
8 restraints
H atoms treated by a mixture of independent and constrained refinement
Δρ_max_ = 0.80 e Å^−3^
Δρ_min_ = −0.53 e Å^−3^

### 

Data collection: *CrysAlis PRO* (Oxford Diffraction, 2007 ▶); cell refinement: *CrysAlis PRO*; data reduction: *CrysAlis RED* (Oxford Diffraction, 2007 ▶); program(s) used to solve structure: *SHELXS97* (Sheldrick, 2008 ▶); program(s) used to refine structure: *SHELXL97* (Sheldrick, 2008 ▶); molecular graphics: *SHELXTL* (Sheldrick, 2008 ▶) and *DIAMOND* (Brandenburg, 1998 ▶); software used to prepare material for publication: *SHELXTL* and *publCIF (*Westrip, 2010 ▶).

## Supplementary Material

Crystal structure: contains datablock(s) I, global. DOI: 10.1107/S1600536811055358/su2346sup1.cif

Structure factors: contains datablock(s) I. DOI: 10.1107/S1600536811055358/su2346Isup2.hkl

Additional supplementary materials:  crystallographic information; 3D view; checkCIF report

## Figures and Tables

**Table 1:** Hydrogen-bond geometry (Å, °)

*D*—H⋯*A*	*D*—H	H⋯*A*	*D*⋯*A*	*D*—H⋯*A*
O1*W*—H1*WA*⋯O2*W*	0.86 (2)	2.37 (4)	3.121 (7)	146 (6)
O1*W*—H1*WB*⋯O2^i^	0.86 (2)	2.28 (5)	2.981 (6)	139 (6)
O1*W*—H1*WB*⋯O5^ii^	0.86 (2)	2.40 (4)	3.084 (6)	137 (5)
O7—H7*O*⋯O3^iii^	0.79 (2)	2.38 (2)	3.174 (5)	179 (5)
O2*W*—H2*WA*⋯O3*W*^iv^	0.64	2.30	2.776 (18)	134
O3*W*—H3*WA*⋯O2*W*^v^	0.86	1.94	2.776 (18)	164
O3*W*—H3*WB*⋯O4^vi^	0.90	2.24	2.811 (15)	121

Symmetry codes: (i) 
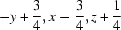
; (ii) 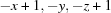; (iii) 
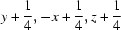
; (iv) 
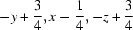
; (v) 
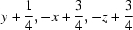
; (vi) 
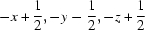
.

**Fig. 1.**
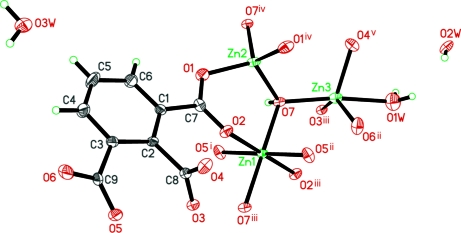
A view of the asymmetric unit of the title compound, showing the coordination environments of the three ZnII centers and the 30% probability displacement ellipsoids [symmetry codes: (i) y + 1/4, -x + 1/4, z + 1/4; (ii) -y + 3/4, x - 1/4,- z + 3/4; (iii) -x + 1, -y, -z + 1; (iv) -x + 1, -y - 1/2, z; (v) -y + 3/4, x - 3/4, z + 1/4].

**Fig. 2.**
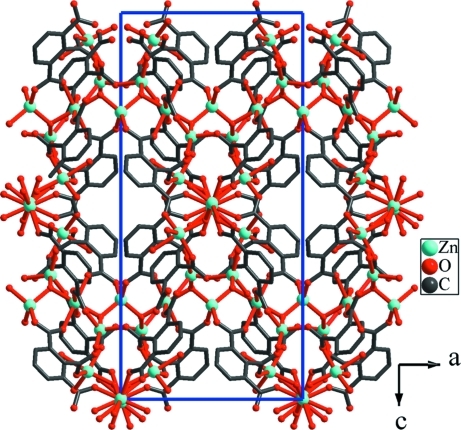
A view along the b axis of the three-dimensional structure of the title compound [the solvent water molecules and the H atoms have been omitted for clarity].

## supplementary crystallographic information

### Comment

The title compound consists of three crystallographically unique Zn^II^ cations, two of which are located on special positions, one benzene-1,2,3-tricarboxylate anion, an OH^-^ anion (O7), a coordinated water molecule (O1W), and two disordered solvent water molecules (Fig. 1).

Atom Zn1 is located on an inversion center and is six coordinated by four oxygen atoms from four symmetry related 1,2,3-tricarboxybenzene anions and two symmetry related OH^-^ anions. The Zn1—*O*(carboxylate) distances are 2.022 (4) and 2.199 (3) Å. The Zn1—O7(OH^-^) distance is 2.089 (3) Å. Atom Zn2, also located on a 2-fold axis, is four coordinated by two oxygen atoms from two symmetry related 1,2,3-tricarboxybenzene anions and two symmetry related OH^-^ anions. The Zn2—O (carboxylate) and Zn2—O7 distances are 1.943 (4) Å and 1.948 (3) Å, respectively. Atom Zn3, locate in a general position, is five coordinated by three oxygen atoms from three different 1,2,3-tricarboxybenzene anions, one OH^-^ anion and one water molecule (O1W). The Zn3—*O*(carboxylate) distances are 1.935 (4), 1.984 (3) and 2.008 (4) Å. The Zn3—O7 distance is 2.069 (4) Å, and the Zn3—O1W distance is 2.136 (5) Å. The Zn—O (carboxylate) distances are similar to those observed in related structures (Wu *et al.*, 2009).

Each benzene-1,2,3-tricarboxylate anion bridges six Zn^II^ centers, and the OH^-^ anion bridges three Zn^II^ centers, leading to the formation of an infinite three-dimensional framework (Fig. 2). A series of O—H···O hydrogen bonds (Table 1) involving the tricarboxybenzene anions, the OH^-^ anion and the coordinating and two solvent water molecules (both of which are only partially occupied), further stabilize the crystal structure (Table 1).

### Experimental

A mixture of benzene-1,2,3-tricarboxylic acid (0.063 g, 0.3 mmol), NaOH (0.036 g, 0.9 mmol), and Zn(Ac)_2_ (0.066 g, 0.3 mmol), in 10 ml H_2_O was sealed in 18 ml Teflon-lined stainless steel container. The container was heated to 433 K and held at that temperature for 72 h. It was then cooled to room temperature at a rate of 10 K per hour and block-like colourless crystals of the title compound were isolated.

### Refinement

C-bound H-atoms were included in calculated positions and were refined as riding atoms: C—H = 0.93 Å, with *U*_iso_ = 1.2*U*_eq_ (C). The OH^-^ and water H atoms were located in difference Fourier maps and were refined with distance restraints of 0.86 (2) Å, or treated as riding atoms, all with *U*_iso_(H) = 1.2*U*_eq_(O). Water molecule O2W located on a 2-fold axis is 0.5 occupied, while water O3W is located in a general position is 0.25 occupied.

### Crystal data

**Table:**

[Zn_2_(C_9_H_3_O_6_)(OH)(H_2_O)]·0.5H_2_O	*D*_x_ = 2.257 Mg m^−^^3^
*M**_r_* = 381.89	Mo *K*α radiation, λ = 0.71073 Å
Tetragonal, *I*4_1_/*a*	Cell parameters from 2048 reflections
Hall symbol: -I 4ad	θ = 2.8–25.3°
*a* = 12.8412 (2) Å	µ = 4.31 mm^−^^1^
*c* = 27.2647 (7) Å	*T* = 293 K
*V* = 4495.85 (15) Å^3^	Block, colourless
*Z* = 16	0.28 × 0.23 × 0.21 mm
*F*(000) = 3024	

### Data collection

**Table:**

Oxford Diffraction Gemini R Ultra diffractometer	2048 independent reflections
Radiation source: fine-focus sealed tube	1402 reflections with *I* > 2σ(*I*)
graphite	*R*_int_ = 0.051
Detector resolution: 10.0 pixels mm^-1^	θ_max_ = 25.3°, θ_min_ = 2.8°
ω scan	*h* = −15→15
Absorption correction: multi-scan (*CrysAlis RED*; Oxford Diffraction, 2007)	*k* = −15→15
*T*_min_ = 0.312, *T*_max_ = 0.399	*l* = −22→32
9288 measured reflections	

### Refinement

**Table:**

Refinement on *F*^2^	Primary atom site location: structure-invariant direct methods
Least-squares matrix: full	Secondary atom site location: difference Fourier map
*R*[*F*^2^ > 2σ(*F*^2^)] = 0.035	Hydrogen site location: inferred from neighbouring sites
*wR*(*F*^2^) = 0.090	H atoms treated by a mixture of independent and constrained refinement
*S* = 0.94	*w* = 1/[σ^2^(*F*_o_^2^) + (0.0506*P*)^2^] where *P* = (*F*_o_^2^ + 2*F*_c_^2^)/3
2048 reflections	(Δ/σ)_max_ < 0.001
198 parameters	Δρ_max_ = 0.80 e Å^−^^3^
8 restraints	Δρ_min_ = −0.53 e Å^−^^3^

### Special details

**Table:**

Geometry. All e.s.d.'s (except the e.s.d. in the dihedral angle between two l.s. planes) are estimated using the full covariance matrix. The cell e.s.d.'s are taken into account individually in the estimation of e.s.d.'s in distances, angles and torsion angles; correlations between e.s.d.'s in cell parameters are only used when they are defined by crystal symmetry. An approximate (isotropic) treatment of cell e.s.d.'s is used for estimating e.s.d.'s involving l.s. planes.
Refinement. Refinement of *F*^2^ against ALL reflections. The weighted *R*-factor *wR* and goodness of fit *S* are based on *F*^2^, conventional *R*-factors *R* are based on *F*, with *F* set to zero for negative *F*^2^. The threshold expression of *F*^2^ > σ(*F*^2^) is used only for calculating *R*-factors(gt) *etc*. and is not relevant to the choice of reflections for refinement. *R*-factors based on *F*^2^ are statistically about twice as large as those based on *F*, and *R*- factors based on ALL data will be even larger.

### Fractional atomic coordinates and isotropic or equivalent isotropic displacement parameters (Å^2^)

**Table:**

	*x*	*y*	*z*	*U*_iso_*/*U*_eq_	Occ. (<1)
Zn1	0.5000	0.0000	0.5000	0.0208 (2)	
Zn2	0.5000	−0.2500	0.49263 (3)	0.0250 (3)	
Zn3	0.68168 (5)	−0.13663 (5)	0.57097 (2)	0.0274 (2)	
O1	0.3829 (3)	−0.2239 (3)	0.44919 (13)	0.0350 (10)	
O1W	0.8258 (4)	−0.1142 (4)	0.61415 (16)	0.0569 (13)	
O2	0.4505 (3)	−0.0690 (3)	0.42997 (12)	0.0275 (9)	
O3	0.3919 (3)	0.1070 (3)	0.36657 (11)	0.0224 (8)	
O4	0.4706 (3)	0.0068 (3)	0.31306 (13)	0.0326 (10)	
O5	0.2600 (3)	0.1066 (3)	0.28024 (12)	0.0312 (9)	
O6	0.1945 (3)	−0.0076 (3)	0.22700 (13)	0.0411 (11)	
O7	0.5375 (3)	−0.1373 (3)	0.53706 (12)	0.0209 (8)	
C1	0.3132 (4)	−0.1383 (4)	0.38044 (18)	0.0250 (13)	
C2	0.3150 (4)	−0.0597 (4)	0.34414 (17)	0.0201 (12)	
C3	0.2429 (4)	−0.0677 (4)	0.30533 (19)	0.0273 (13)	
C4	0.1747 (5)	−0.1498 (5)	0.3023 (2)	0.0485 (18)	
H4	0.1296	−0.1543	0.2757	0.058*	
C5	0.1720 (6)	−0.2254 (5)	0.3380 (3)	0.061 (2)	
H5	0.1251	−0.2803	0.3357	0.074*	
C6	0.2400 (5)	−0.2187 (5)	0.3774 (2)	0.0439 (17)	
H6	0.2370	−0.2685	0.4022	0.053*	
C7	0.3880 (4)	−0.1430 (4)	0.42282 (17)	0.0239 (12)	
C8	0.3973 (4)	0.0242 (4)	0.34212 (17)	0.0197 (12)	
C9	0.2329 (4)	0.0172 (4)	0.26799 (19)	0.0246 (12)	
O2W	1.0000	−0.2500	0.5664 (4)	0.038 (3)	0.50
H2WA	0.9977	−0.2203	0.5475	0.057*	0.50
O3W	0.0743 (12)	−0.3649 (12)	0.2625 (5)	0.038 (4)	0.25
H3WA	0.0608	−0.3205	0.2396	0.057*	0.25
H3WB	0.0318	−0.4194	0.2582	0.057*	0.25
H1WA	0.885 (3)	−0.124 (5)	0.6006 (18)	0.057*	
H1WB	0.825 (4)	−0.141 (5)	0.6431 (11)	0.057*	
H7O	0.493 (3)	−0.139 (4)	0.5572 (14)	0.019 (16)*	

### Atomic displacement parameters (Å^2^)

**Table:**

	*U*^11^	*U*^22^	*U*^33^	*U*^12^	*U*^13^	*U*^23^
Zn1	0.0265 (5)	0.0185 (5)	0.0174 (4)	−0.0003 (4)	0.0020 (4)	0.0024 (3)
Zn2	0.0434 (6)	0.0173 (5)	0.0143 (4)	−0.0014 (4)	0.000	0.000
Zn3	0.0421 (5)	0.0242 (4)	0.0159 (3)	−0.0009 (3)	0.0057 (3)	0.0027 (3)
O1	0.047 (3)	0.030 (2)	0.028 (2)	−0.007 (2)	−0.0119 (19)	0.0136 (18)
O1W	0.054 (3)	0.073 (4)	0.043 (3)	0.000 (3)	−0.003 (2)	0.007 (3)
O2	0.040 (2)	0.028 (2)	0.0146 (18)	−0.0088 (19)	−0.0061 (16)	0.0001 (15)
O3	0.031 (2)	0.021 (2)	0.0149 (18)	−0.0029 (17)	−0.0013 (15)	0.0002 (15)
O4	0.036 (2)	0.037 (2)	0.024 (2)	−0.0105 (19)	0.0064 (18)	−0.0061 (17)
O5	0.047 (3)	0.024 (2)	0.0218 (19)	−0.0069 (19)	−0.0138 (18)	0.0090 (16)
O6	0.062 (3)	0.033 (2)	0.028 (2)	0.010 (2)	−0.025 (2)	−0.0059 (18)
O7	0.034 (2)	0.018 (2)	0.0101 (18)	−0.0017 (17)	0.0051 (17)	0.0005 (15)
C1	0.029 (3)	0.021 (3)	0.025 (3)	0.003 (3)	−0.006 (2)	0.000 (2)
C2	0.028 (3)	0.016 (3)	0.016 (3)	0.001 (2)	−0.002 (2)	−0.005 (2)
C3	0.033 (3)	0.021 (3)	0.028 (3)	0.002 (3)	−0.009 (3)	−0.002 (2)
C4	0.059 (5)	0.035 (4)	0.052 (4)	−0.009 (3)	−0.035 (4)	0.008 (3)
C5	0.065 (5)	0.041 (4)	0.078 (5)	−0.029 (4)	−0.037 (4)	0.020 (4)
C6	0.051 (4)	0.032 (4)	0.049 (4)	−0.009 (3)	−0.024 (3)	0.016 (3)
C7	0.025 (3)	0.033 (3)	0.013 (3)	0.005 (3)	0.003 (2)	0.000 (2)
C8	0.027 (3)	0.021 (3)	0.011 (2)	−0.001 (2)	−0.003 (2)	0.004 (2)
C9	0.023 (3)	0.026 (3)	0.025 (3)	0.001 (3)	−0.008 (2)	−0.001 (2)
O2W	0.039 (7)	0.030 (7)	0.044 (7)	−0.018 (5)	0.000	0.000
O3W	0.044 (11)	0.037 (10)	0.033 (9)	0.004 (8)	0.010 (8)	−0.014 (7)

### Geometric parameters (Å, °)

**Table:**

Zn1—O5^i^	2.022 (4)	O4—Zn3^vi^	2.008 (4)
Zn1—O5^ii^	2.022 (4)	O5—C9	1.245 (6)
Zn1—O7^iii^	2.089 (3)	O5—Zn1^vii^	2.022 (3)
Zn1—O7	2.089 (3)	O6—C9	1.263 (6)
Zn1—O2	2.199 (3)	O6—Zn3^viii^	1.935 (4)
Zn1—O2^iii^	2.199 (3)	O7—H7O	0.79 (2)
Zn2—O1^iv^	1.943 (4)	C1—C6	1.399 (8)
Zn2—O1	1.943 (4)	C1—C2	1.413 (7)
Zn2—O7	1.948 (3)	C1—C7	1.504 (7)
Zn2—O7^iv^	1.948 (3)	C2—C3	1.410 (7)
Zn3—O6^i^	1.935 (4)	C2—C8	1.511 (7)
Zn3—O3^iii^	1.984 (3)	C3—C4	1.373 (8)
Zn3—O4^v^	2.008 (4)	C3—C9	1.497 (7)
Zn3—O7	2.069 (4)	C4—C5	1.374 (9)
Zn3—O1W	2.213 (5)	C4—H4	0.9300
O1—C7	1.265 (6)	C5—C6	1.388 (9)
O1W—H1WA	0.86 (2)	C5—H5	0.9300
O1W—H1WB	0.86 (2)	C6—H6	0.9300
O2—C7	1.259 (6)	O2W—H2WA	0.6405
O3—C8	1.257 (6)	O3W—H3WA	0.8614
O3—Zn3^iii^	1.984 (3)	O3W—H3WB	0.8960
O4—C8	1.250 (6)		
			
O5^i^—Zn1—O5^ii^	180.000 (1)	C8—O4—Zn3^vi^	123.3 (3)
O5^i^—Zn1—O7^iii^	87.68 (15)	C9—O5—Zn1^vii^	135.5 (3)
O5^ii^—Zn1—O7^iii^	92.32 (14)	C9—O6—Zn3^viii^	133.0 (4)
O5^i^—Zn1—O7	92.32 (14)	Zn2—O7—Zn3	120.15 (18)
O5^ii^—Zn1—O7	87.68 (15)	Zn2—O7—Zn1	105.62 (15)
O7^iii^—Zn1—O7	180.00 (15)	Zn3—O7—Zn1	114.77 (16)
O5^i^—Zn1—O2	86.24 (15)	Zn2—O7—H7O	104 (4)
O5^ii^—Zn1—O2	93.76 (15)	Zn3—O7—H7O	110 (4)
O7^iii^—Zn1—O2	81.58 (13)	Zn1—O7—H7O	101 (4)
O7—Zn1—O2	98.42 (13)	C6—C1—C2	119.8 (5)
O5^i^—Zn1—O2^iii^	93.76 (15)	C6—C1—C7	116.4 (5)
O5^ii^—Zn1—O2^iii^	86.24 (15)	C2—C1—C7	123.8 (5)
O7^iii^—Zn1—O2^iii^	98.42 (13)	C3—C2—C1	117.6 (5)
O7—Zn1—O2^iii^	81.58 (13)	C3—C2—C8	118.9 (4)
O2—Zn1—O2^iii^	180.000 (1)	C1—C2—C8	123.1 (4)
O1^iv^—Zn2—O1	104.9 (2)	C4—C3—C2	121.3 (5)
O1^iv^—Zn2—O7	108.41 (15)	C4—C3—C9	117.7 (5)
O1—Zn2—O7	116.25 (16)	C2—C3—C9	120.9 (5)
O1^iv^—Zn2—O7^iv^	116.25 (16)	C3—C4—C5	121.1 (6)
O1—Zn2—O7^iv^	108.41 (15)	C3—C4—H4	119.5
O7—Zn2—O7^iv^	103.1 (2)	C5—C4—H4	119.5
O6^i^—Zn3—O3^iii^	135.77 (17)	C4—C5—C6	119.3 (6)
O6^i^—Zn3—O4^v^	102.75 (17)	C4—C5—H5	120.4
O3^iii^—Zn3—O4^v^	117.03 (14)	C6—C5—H5	120.4
O6^i^—Zn3—O7	98.68 (14)	C5—C6—C1	120.9 (6)
O3^iii^—Zn3—O7	87.62 (14)	C5—C6—H6	119.5
O4^v^—Zn3—O7	107.46 (15)	C1—C6—H6	119.5
O6^i^—Zn3—O1W	82.42 (17)	O2—C7—O1	124.3 (5)
O3^iii^—Zn3—O1W	85.21 (16)	O2—C7—C1	119.8 (5)
O4^v^—Zn3—O1W	81.20 (18)	O1—C7—C1	115.9 (5)
O7—Zn3—O1W	170.65 (17)	O4—C8—O3	122.0 (5)
C7—O1—Zn2	116.6 (3)	O4—C8—C2	115.0 (4)
Zn3—O1W—H1WA	120 (4)	O3—C8—C2	123.0 (5)
Zn3—O1W—H1WB	116 (4)	O5—C9—O6	125.4 (5)
H1WA—O1W—H1WB	110 (3)	O5—C9—C3	117.7 (4)
C7—O2—Zn1	128.5 (3)	O6—C9—C3	116.8 (5)
C8—O3—Zn3^iii^	130.1 (3)	H3WA—O3W—H3WB	107.3
			
O1^iv^—Zn2—O1—C7	−65.4 (4)	C7—C1—C2—C8	4.3 (8)
O7—Zn2—O1—C7	54.3 (4)	C1—C2—C3—C4	−1.2 (9)
O7^iv^—Zn2—O1—C7	169.8 (4)	C8—C2—C3—C4	172.1 (6)
O5^i^—Zn1—O2—C7	141.0 (4)	C1—C2—C3—C9	174.2 (5)
O5^ii^—Zn1—O2—C7	−39.0 (4)	C8—C2—C3—C9	−12.5 (8)
O7^iii^—Zn1—O2—C7	−130.8 (5)	C2—C3—C4—C5	2.2 (11)
O7—Zn1—O2—C7	49.2 (5)	C9—C3—C4—C5	−173.4 (7)
O2^iii^—Zn1—O2—C7	−117 (100)	C3—C4—C5—C6	−0.5 (12)
O1^iv^—Zn2—O7—Zn3	−45.2 (2)	C4—C5—C6—C1	−2.0 (12)
O1—Zn2—O7—Zn3	−163.02 (17)	C2—C1—C6—C5	2.9 (10)
O7^iv^—Zn2—O7—Zn3	78.55 (17)	C7—C1—C6—C5	−175.8 (6)
O1^iv^—Zn2—O7—Zn1	86.51 (19)	Zn1—O2—C7—O1	−39.5 (7)
O1—Zn2—O7—Zn1	−31.3 (2)	Zn1—O2—C7—C1	140.8 (4)
O7^iv^—Zn2—O7—Zn1	−149.7 (2)	Zn2—O1—C7—O2	−15.6 (7)
O6^i^—Zn3—O7—Zn2	91.9 (2)	Zn2—O1—C7—C1	164.2 (3)
O3^iii^—Zn3—O7—Zn2	−132.1 (2)	C6—C1—C7—O2	−174.5 (5)
O4^v^—Zn3—O7—Zn2	−14.5 (2)	C2—C1—C7—O2	6.8 (8)
O1W—Zn3—O7—Zn2	−172.0 (9)	C6—C1—C7—O1	5.7 (8)
O6^i^—Zn3—O7—Zn1	−35.8 (2)	C2—C1—C7—O1	−172.9 (5)
O3^iii^—Zn3—O7—Zn1	100.22 (18)	Zn3^vi^—O4—C8—O3	27.5 (7)
O4^v^—Zn3—O7—Zn1	−142.15 (16)	Zn3^vi^—O4—C8—C2	−154.5 (3)
O1W—Zn3—O7—Zn1	60.3 (10)	Zn3^iii^—O3—C8—O4	−156.6 (4)
O5^i^—Zn1—O7—Zn2	−90.46 (18)	Zn3^iii^—O3—C8—C2	25.5 (7)
O5^ii^—Zn1—O7—Zn2	89.54 (18)	C3—C2—C8—O4	−75.5 (6)
O7^iii^—Zn1—O7—Zn2	118.7 (5)	C1—C2—C8—O4	97.4 (6)
O2—Zn1—O7—Zn2	−3.92 (19)	C3—C2—C8—O3	102.5 (6)
O2^iii^—Zn1—O7—Zn2	176.08 (19)	C1—C2—C8—O3	−84.5 (7)
O5^i^—Zn1—O7—Zn3	44.24 (18)	Zn1^vii^—O5—C9—O6	5.9 (9)
O5^ii^—Zn1—O7—Zn3	−135.76 (18)	Zn1^vii^—O5—C9—C3	−171.8 (4)
O7^iii^—Zn1—O7—Zn3	−106.6 (3)	Zn3^viii^—O6—C9—O5	−27.8 (9)
O2—Zn1—O7—Zn3	130.78 (17)	Zn3^viii^—O6—C9—C3	149.8 (4)
O2^iii^—Zn1—O7—Zn3	−49.22 (17)	C4—C3—C9—O5	151.0 (6)
C6—C1—C2—C3	−1.3 (8)	C2—C3—C9—O5	−24.6 (8)
C7—C1—C2—C3	177.3 (5)	C4—C3—C9—O6	−26.8 (8)
C6—C1—C2—C8	−174.3 (5)	C2—C3—C9—O6	157.6 (5)

Symmetry codes: (i) −*y*+3/4, *x*−1/4, −*z*+3/4; (ii) *y*+1/4, −*x*+1/4, *z*+1/4; (iii) −*x*+1, −*y*, −*z*+1; (iv) −*x*+1, −*y*−1/2, *z*; (v) −*y*+3/4, *x*−3/4, *z*+1/4; (vi) *y*+3/4, −*x*+3/4, *z*−1/4; (vii) −*y*+1/4, *x*−1/4, *z*−1/4; (viii) *y*+1/4, −*x*+3/4, −*z*+3/4.

### Hydrogen-bond geometry (Å, °)

**Table:**

*D*—H···*A*	*D*—H	H···*A*	*D*···*A*	*D*—H···*A*
O1W—H1WA···O2W	0.86 (2)	2.37 (4)	3.121 (7)	146 (6)
O1W—H1WB···O2^v^	0.86 (2)	2.28 (5)	2.981 (6)	139 (6)
O1W—H1WB···O5^iii^	0.86 (2)	2.40 (4)	3.084 (6)	137 (5)
O7—H7O···O3^ii^	0.79 (2)	2.38 (2)	3.174 (5)	179 (5)
O2W—H2WA···O3W^i^	0.64	2.30	2.776 (18)	134
O3W—H3WA···O2W^viii^	0.86	1.94	2.776 (18)	164
O3W—H3WB···O4^ix^	0.90	2.24	2.811 (15)	121

Symmetry codes: (v) −*y*+3/4, *x*−3/4, *z*+1/4; (iii) −*x*+1, −*y*, −*z*+1; (ii) *y*+1/4, −*x*+1/4, *z*+1/4; (i) −*y*+3/4, *x*−1/4, −*z*+3/4; (viii) *y*+1/4, −*x*+3/4, −*z*+3/4; (ix) −*x*+1/2, −*y*−1/2, −*z*+1/2.

## References

[bb1] Brandenburg, K. (1998). *DIAMOND* Crystal Impact GbR, Bonn, Germany.

[bb2] Chui, S. S. Y., Siu, A. & Williams, I. D. (1999). *Acta Cryst.* C**55**, 194–196.

[bb3] Majumder, A., Shit, S., Choudhury, C. R., Batten, S. R., Pilet, G., Daro, N., Sutter, J.-P., Chattopadhyay, N. & Mitra, S. (2005). *Inorg. Chim. Acta*, **358**, 3855–3864.

[bb4] Oxford Diffraction (2007). *CrysAlis PRO* and *CrysAlis RED* Oxford Diffraction Ltd, Abingdon, England.

[bb5] Sheldrick, G. M. (2008). *Acta Cryst.* A**64**, 112–122. 10.1107/S0108767307043930 18156677

[bb6] Westrip, S. P. (2010). *J. Appl. Cryst.* **43**, 920–925.

[bb7] Wu, H., Zhang, L. P., Liu, H. Y., Yang, J. & Ma, J. F. (2009). *Sci. China Ser. B Chem.* **52**, 1490.

